# Modulatory effects of black jujube melanoidins on gut microbiota and metabolic pathways in high-fat diet-induced obesity

**DOI:** 10.3389/fnut.2025.1580439

**Published:** 2025-05-06

**Authors:** Xi Che, Yifei Zhao, Yuxiao Wang, Xin Sun, Hongxun Tao, Rentang Zhang

**Affiliations:** ^1^College of Food Science and Engineering, Shandong Agricultural University, Tai'an, Shandong, China; ^2^College of Food and Biological Engineering, Jiangsu University, Zhenjiang, China

**Keywords:** black jujube, melanoidin, obesity, gut microbiota, metabolomics

## Abstract

**Introduction:**

Obesity, a growing public health issue, is closely associated with excessive energy intake and gut microbiota imbalances. Despite the increasing attention given to black jujube as a blackened fermented food in recent years, the role of its melanoidins (MLD) in obesity-related biological mechanisms has yet to be thoroughly investigated.

**Methods:**

This study investigated the effects of black jujube MLD on obesity induced by a high-fat diet in C57BL/6J mice. We hypothesize that MLD exerts an anti-obesity effect, potentially mediated by alterations in gut microbiota composition and the modulation of metabolic responses.

**Results:**

The results demonstrate that MLD administration mitigated HFD-induced weight gain, improved insulin resistance, and enhanced glucose metabolism by reducing blood glucose and insulin levels (*p* < 0.05). MLD also protected the liver, as evidenced by a 16.4% decrease in alanine aminotransferase (ALT) levels and a 29.6% decrease in aspartate aminotransferase (AST) levels (*p* < 0.05). Furthermore, MLD treatment restored the compromised intestinal microbiota to a healthier state at the genus level by lowering the *Firmicutes*/*Bacteroidetes* ratio (38.9%), suppressing the abundance of pathogenic bacteria, such as *Erysipelatoclostridium* and *Bacteroides* descendants, and promoting the growth of beneficial bacteria, including *Bifidobacterium and norank_f_Muribaculaceae*. Metabolomic profiling revealed that MLD can alleviate metabolic disorders by downregulating arginine metabolism and sphingolipid metabolism. Its metabolites are significantly correlated with several bacterial genera, including *Bacteroides, Roseburia, Erysipelatoclostridium, Bacteroides, norank_f_Muribaculaceae*, and *Lachnospiraceae NK4A136 group*.

**Discussion:**

Based on these findings, MLD may mitigate obesity and other associated metabolic disorders by modulating the gut microbiome–metabolism axis.

## 1 Introduction

Obesity is a critical public health challenge that plays a central role in the development of metabolic disorders, such as non-alcoholic fatty liver disease, insulin resistance, and hyperglycemia (1). It is primarily driven by an imbalance between caloric intake and energy expenditure (2). When energy intake surpasses the body's requirements, the excess is stored as fat and glycogen in subcutaneous adipose tissue and various organs, leading to obesity. The gut microbiota composition has a critical influence on the body's ability to absorb nutrients and regulate energy metabolism. As such, it plays a fundamental role in the onset and progression of obesity and its associated metabolic disorders (3). Studies have demonstrated that obesity and related metabolic disorders are frequently accompanied by significant gut dysbiosis, which promotes increased bacterial energy extraction from the gastrointestinal tract (4). Moreover, changes in the abundance of obesity-associated microbiota, such as *Bifidobacterium bifidum* and *Bifidobacterium paracasei*, have been shown to influence fat deposition, glucose tolerance, systemic inflammation, and dyslipidemia in the host. Several lines of evidence suggest that certain gut bacteria and their metabolites may offer protective effects against obesity and related metabolic dysfunction (3), potentially serving as therapeutic targets in the management of these conditions.

Numerous studies have highlighted the modulatory effects of melanoidins on the intestinal microbiota in mice. The gastrointestinal tract is regarded as the primary site for the biological activity of melanoidins, where they primarily function as dietary fibers and prebiotics (5). These compounds act as potent free radical scavengers and play a crucial role in regulating gut microbiota composition (6). Research consistently demonstrates that melanoidins exert beneficial effects on intestinal flora. For instance, varying molecular weights of black garlic melanoidins have been shown to mitigate dysbiosis induced by high-fat diets. Specifically, high-molecular-weight melanoidins exert a significant impact on both lipid metabolism and the modulation of gut microbiota in mice (7). High doses of barley malt-derived melanoidins have been shown to induce significant alterations in the gut microbiota, stimulating the production of short-chain fatty acids (SCFAs) and enhancing the relative abundance of beneficial bacteria, such as *Lactobacillus* and *Umbelliferae* (8). Furthermore, a study simulating gastrointestinal digestion revealed that melanoidins in cookie skins fostered SCFA production by gut microbes (9). Blackened jujube, a novel product obtained through high-temperature, high-humidity processing of red jujubes, is recognized for its rich taste and nutritional value (10). As Maillard reaction products, melanoidins play a pivotal role in modulating intestinal flora. However, despite this extensive research, there was limited literature addressing the specific impact of melanoidins on obesity.

The key roles of melanoidins in flavor, texture, and bioactivity are intricately influenced by physical alterations and structural transformations (11). As a fermented food product that darkens under controlled temperature and humidity, black jujube may contain melanoidins with distinct chemical structures, molecular weights, and biological activities compared to those in other commonly consumed foods. While previous studies have demonstrated the antioxidant, anti-inflammatory, and gut microbiota-modulating potential of melanoidins, systematic investigations into the effects of black jujube melanoidins on high-fat diet-induced obesity and metabolic pathways remain limited. We hypothesize that melanoidins extracted from black jujube have anti-obesity effects, potentially by influencing gut microbiota composition and modulating metabolic responses. To evaluate this hypothesis, we established an obese mouse model induced by a high-fat diet and examined the effects of MLD through biochemical analysis, H&E staining, gut microbiome profiling, and metabolomics techniques. The study aims to elucidate the relationship between MLD, the gut microbiome, metabolomics, and obesity, providing a strategic framework for the development of novel functional products targeting obesity.

## 2 Materials and methods

### 2.1 Materials

Z. jujuba cv. Jinsixiaozao (Golden Silk Jujubes) was purchased from Guizitang Food Science and Technology Co., Ltd. (Shandong, China). X-5 Macroporous Resin was provided by Shanghai Yuanye Biotechnology Co., Ltd. (Shanghai, China). Hydrochloric acid was obtained from Tianjin Komeo Chemical Reagent Co., Ltd. (Tianjin, China). ELISA kits for total cholesterol (TC), triglycerides (TG), low-density lipoprotein cholesterol (LDL-C), high-density lipoprotein cholesterol (HDL-C), ALT, and AST were obtained from Shanghai Enzyme-linked Biotechnology Co., Ltd. (Shanghai, China). Unless otherwise noted, all chemicals and reagents used in this study were of analytical grade.

### 2.2 Extraction and separation of MLD samples from blackened jujube

The extraction of MLD was conducted following a previously established procedure with slight modifications (12, 13). Z. jujuba cv. Jinsixiaozao was subjected to spontaneous fermentation at 65°C for 84 h. The blackened jujube juice was then extracted at 80°C for 100 min, using a jujube-to-water ratio of 1:4. The resulting extract was filtered twice through medium-speed qualitative filter paper. The filtrate was adsorbed onto X-5 macroporous resin and subsequently eluted with a 60% ethanol solution. The combined eluate was collected as the MLD solution and lyophilized. The MLD was reconstituted into a 100 mg/mL solution and processed through an ultrafiltration system (BT100-1F, Baoding Longer Precision Pump Co., Ltd., Hebei, China) with a molecular weight cut-off of 50 kDa. The solution was washed five times through the membrane to isolate MLD (>50 kDa). Finally, the collected MLD solutions were lyophilized and stored at −20°C for future use.

### 2.3 Fourier transform infrared spectroscopy

The Fourier transform infrared (FTIR) analysis of melanoidins was performed according to the previously reported method (14). A 2 mg sample of black jujube-like melanoidins was compressed into tablets with potassium bromide and analyzed throughout a wavelength range of 4,000–400 cm^−1^.

### 2.4 Molecular weight distribution analysis

Molecular weight distribution analysis of melanoidins was performed with slight modifications to the method described by Lu et al. (15). The analysis was performed using an Agilent PL-GPC50 column, with a sample concentration of 0.10 mg/mL, an injection volume of 200 μL, and water as the mobile phase.

### 2.5 Animal experiments

Seven-week-old male C57BL/6J mice (specific pathogen-free grade, *n* = 36, 20 ± 1 g) were obtained from Beijing Viton Lihua Laboratory Animal Technology Co., Ltd. (Beijing, China). All mice were housed under specific pathogen-free (SPF) conditions at a temperature of 23 ± 1°C, a relative humidity of 40–60%, and a 12-h light/dark cycle. After a 1-week acclimation period, the mice were randomly assigned to three groups using an utterly randomized allocation method, including a normal feed control group (NC) (*n* = 12), a high-fat diet group (HFD) (*n* = 12), and a melanoidin treatment group (MLD, 500 mg/kg/d) (*n* = 12) (16). The dose was selected based on effects observed in preliminary trials. The NC group was fed a standard diet (10% kcal energy from fat, 20% protein, and 70% carbohydrates; Cat. # SYCON50J; Shanghai Shuyu Biotechnology Co., Ltd, China), while the other two groups received a high-fat diet (60% kcal energy from fat, 20% protein, and 20% carbohydrates; Cat. # SYHF60; Shanghai Shuyu Biotechnology Co., Ltd, China) for 20 weeks to induce the high-fat model. Following this period, as outlined in Supplementary Table S1, each mouse was administered 0.1 mL/10 g of the respective treatment daily at 9:00 a.m. for 8 weeks (17). This study was conducted in accordance with the ethical guidelines established by the Ethics Committee for Animal Experiments at Shandong Agricultural University (SDAUA-2023-179).

Before euthanasia, fecal samples were collected and stored at−80°C for subsequent analysis. After a 12-h fasting period, the mice were euthanized via cervical dislocation. Blood samples were obtained from the orbital sinus under anesthesia, then centrifuged at 4,000 g for 15 min at 4°C. The resulting supernatant was stored at −80°C until further analysis. Organs, including the heart, liver, spleen, kidneys, perirenal fat, epididymal fat, brown adipose tissue, colon, and empty stomach, were weighed and promptly collected. These tissues were either stored in liquid nitrogen for preservation or fixed in a paraformaldehyde solution at room temperature for subsequent histological analysis.

### 2.6 Assessment of body weight, food intake, OGTT, and ITT

Throughout the experiment, food intake, spillage, and leftover food were carefully recorded, while body weight was measured every week. In 17 and 20 weeks, following a 12-h fast, the mice were gavaged with a glucose dose of 2 g·kg∧-1 body weight (7). In 20 weeks, after another 12-h fasting period, the mice were administered an intraperitoneal injection of human insulin (Novolin R, Novo Nordisk, Bagsværd, Denmark) at a dose of 0.75 IU·kg∧-1 body weight (18). For both the OGTT and the ITT, blood glucose levels were monitored at 0, 15, 30, 60, 90, and 120 min using an Accu-Chek Active glucose meter (Roche, Basel, Switzerland).

### 2.7 Serum biochemical and histological analysis

Serum levels of glucose, TC, TG, LDL-C, HDL-C, ALT, AST, and other liver function-related biomarkers were measured using commercial kits from Shanghai Enzyme-linked Biotechnology Co., Ltd. (Shanghai, China). Liver and adipose tissue samples were stained with H&E according to standard protocols (1, 17). The samples were fixed in a 10% formalin-buffered solution and sectioned into 5 μm slices. All slides were examined using a positive white light photomicrographic microscope (Eclipse Ci-L, Olympus Corp., Japan).

### 2.8 16s rRNA gene sequencing analysis

Total genomic DNA was extracted from fecal samples using the Fast DNA SPIN Extraction Kit (MP Biomedicals, Santa Ana, CA) (19, 20). Purified DNA libraries were sequenced on an Illumina MiSeq PE300 platform (Majorbio Bio-Pharm Technology, China). DNA was subsequently amplified using universal bacterial primers, barcoded 338F (5'-ACTCCTACCTACGGAGGCAGCAGCAG-3') and 806R (5'-GGACTACHVGGGGTWTCTAAT-3'), which target the V3-V4 variable regions of the 16S rRNA gene. The resulting sequences underwent quality filtering with FASTP (v0.19.6) and were merged using FLASH (v1.2.11). High-quality sequences were then denoised with the DADA2 plugin in Qiime2 (v2020.2). The taxonomic classification of amplicon sequence variants (ASVs) was conducted using the Naïve Bayes classifier in Qiime2 with the SILVA 16S rRNA database (v138). Valid sequences were clustered into operational taxonomic units (OTUs) at a 97% sequence similarity threshold using UCLUST and assigned to taxonomic ranks (phylum, order, class, family, and genus).

### 2.9 Untargeted metabolomics analysis

Samples (50 mg) were placed into 2 mL centrifuge tubes containing a 6 mm grinding ball. Next, 400 μL of the extraction solvent (methanol: water, 4:1, v/v) with 0.02 mg/mL internal standard (L-2-chlorophenylalanine, Shanghai Aladdin Biochemical Technology Co., Ltd., Shanghai, China) was added. The tissue was then homogenized for 6 min using a frozen tissue grinder at −10°C and 50 Hz, followed by ultrasonic extraction at 5°C and 40 kHz for 30 min. The sample was stored at −20°C for 30 min and subsequently centrifuged at 4°C for 15 min at 13,000 g. The supernatant was carefully transferred to a sample vial containing an endotractable tube for automated analysis. Additionally, 20 μL of the supernatant from each sample was pooled to create a quality control sample. LC-MS analysis was performed using a UHPLC-Q Exactive HF-X ultra-high-performance liquid chromatography-tandem Fourier transform mass spectrometry system (Thermo Fisher, USA) (21).

### 2.10 Statistical analysis

The data are presented as means ± standard deviation (Mean ± SD). Differences between groups were evaluated using one-way analysis of variance (ANOVA), and *post-hoc* Tukey HSD was subsequently performed using GraphPad Prism 9.5 (GraphPad Software, San Diego, USA) or SPSS Statistics 25.0 (IBM, New York, USA). A significance level of *p* < 0.05 (*n* = 6) was considered statistically significant.

## 3 Results and discussion

### 3.1 Molecular weight distribution analysis

The molecular weight distribution of the melanoidins is shown in Supplementary Figure 1A. MLD exhibits a broad molecular weight distribution, with a predominant concentration in the high molecular weight (HMW) range. Melanoidins with higher molecular weights, typically formed through prolonged fermentation, exhibit greater stability and biological activity than their lower-molecular-weight counterparts (22). It further reveals that MLD is a mixture of highly polymerized components with close molecular connections, where HMW constitutes the majority in black jujube. This distribution aligns with that observed in black garlic melanoidins (23).

### 3.2 FTIR analysis

The FTIR spectrum of MLD is presented in Supplementary Figure 1B. The broad absorption band at 3,409.88 cm^−1^ is assigned to the stretching vibrations of hydroxyl (–OH) groups in polysaccharides, phenolic compounds, and the N–H stretching of primary amines (24). The band at 2,932 cm^−1^ is attributed to C–H stretching vibrations of –CH_2_ and –CH_3_ groups in aliphatic chains (24). The band at 1,738 cm^−1^ indicates the presence of a carbonyl (C=O) group, likely corresponding to ester or carboxyl bonds in MLD (25). A band around 1,633 cm^−1^ is observed, possibly due to O-H bending vibrations or the asymmetric stretching of C=O bonds (amide I band) (26). The absorption band at 1,417 cm^−1^ is likely attributed to C-H bending vibrations (27). The weak band at 1,372 cm^−1^ may result from C-H bending deformations (28). The band at 1,237 cm^−1^ is most likely caused by C-O or C-N (amide III) stretching vibrations in phenolic compounds (28). The band observed at 1,028 cm^−1^ is associated with the C-O-C stretching vibration of the glycosidic bond (29). Overall, the MLD exhibits a protein-carbohydrate structure, similar to ginseng-like melanoidins reported in previous studies (30).

### 3.3 Analyses of body weight, feed intake, OGTT, and ITT

**Figure 1:**
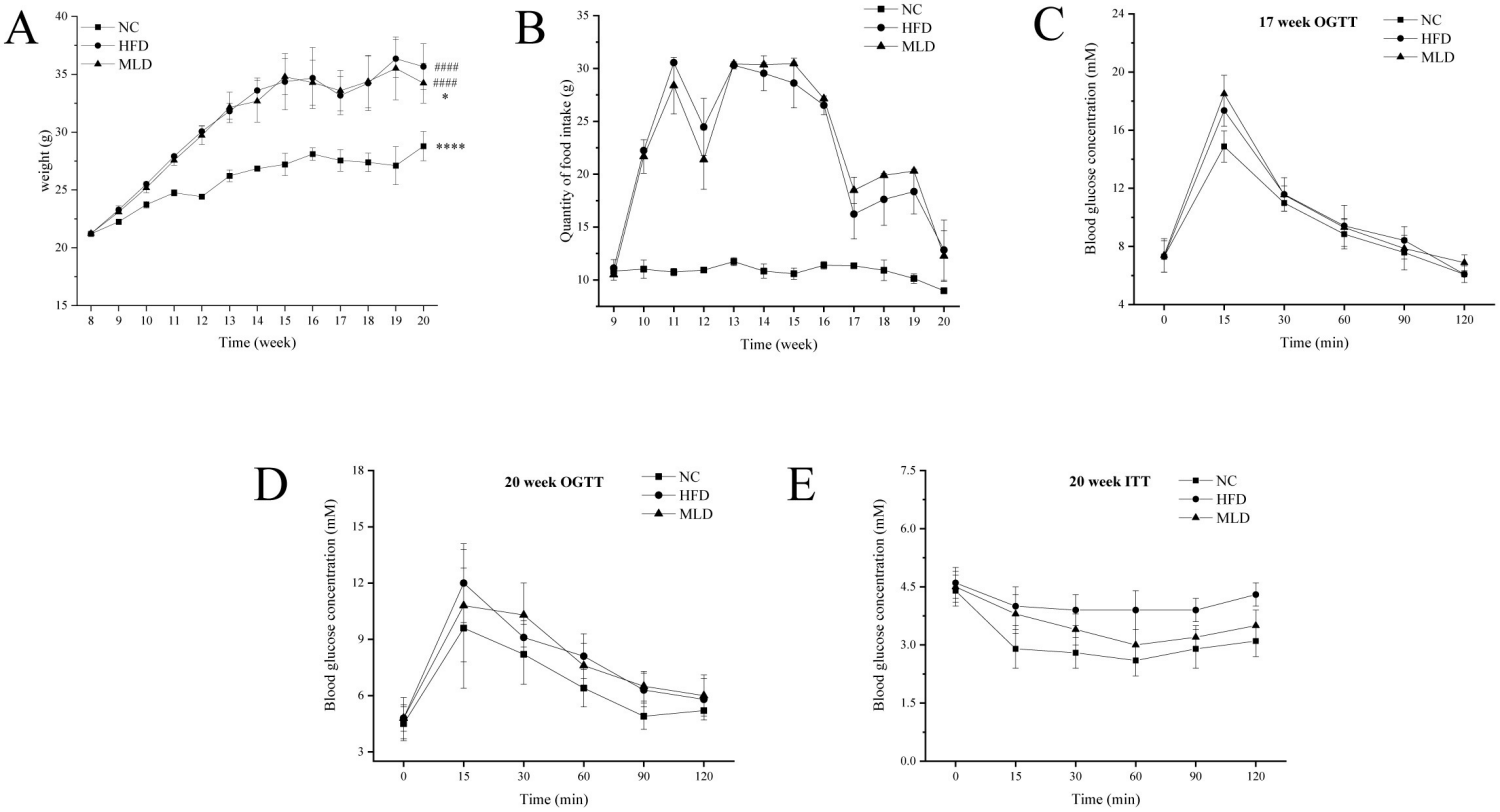
Effects of melanoidins on body weight, feed intake, insulin sensitivity, and glucose tolerance in high-fat mice. **(A)** Weight, **(B)** Feed intake, **(C, D)** OGTT, **(E)** ITT. Values are expressed as means ± SD, *n* = 12. Compared to the NC group, ^*####*^*p* < 0.0001, Compared to the HFD group, ****p < 0.0001, *p < 0.05.

The changes in body weight and food intake across the three experimental groups (NC, HFD, and MLD) over 20 weeks are depicted in Figures 1A, B. The initial body weights of mice in all groups were ~22 g, with no significant differences between the groups. Throughout the study, mice in the MLD group exhibited a higher body weight compared to the NC group, but their body weight remained lower than that of mice in the HFD group (*p* < 0.05). These findings suggest that melanoidin intervention effectively attenuates weight gain induced by a high-fat diet. There were no significant differences in food intake between the groups (*p* > 0.05), which implies that the effect of MLD on body weight was not attributable to reduced food consumption or calorie intake. This is consistent with previous studies on the role of mung beans in weight reduction (31). These results suggest that a high-fat diet induces metabolic disruptions and that MLD can mitigate these effects by reducing both body weight and fat accumulation without altering energy intake (32, 33). Wu et al. (7) demonstrated that various doses of black garlic melanoidins effectively inhibited weight gain, with higher doses showing greater efficacy compared to lower or medium doses (7).

The OGTT and ITT are well-established methods for evaluating the body's ability to regulate blood glucose, serving as critical indicators in the management of obesity (18). The OGTT results showed that all three mouse groups reached their peak blood glucose concentrations at 15 min, followed by a subsequent decline (Figures 1C, D), indicating standard glucose tolerance across all groups. However, compared to the NC group, the HFD and MLD groups exhibited a more rapid rise and a slower decline in blood glucose levels. Notably, mice in the MLD group exhibited a slower increase and a faster reduction in blood glucose levels compared to those in the HFD group, indicating that MLD enhances the body's ability to regulate glucose (Figure 1D). In the ITT, the magnitude of blood glucose reduction following insulin administration serves as an indicator of systemic insulin sensitivity. The ITT results revealed a more pronounced decrease in blood glucose levels in the MLD group (Figure 1E), suggesting that MLD enhances insulin sensitivity in insulin-resistant mice fed a high-fat diet. Collectively, these findings indicate that MLD effectively ameliorates insulin resistance in C57BL/6J mice (17, 32, 33). Similar effects were observed with black garlic melanoidins, which significantly reduced body weight without affecting food intake while also improving insulin and blood glucose regulation in obese mice (17, 34).

### 3.4 Serum biochemical analyses

**Figure 2:**
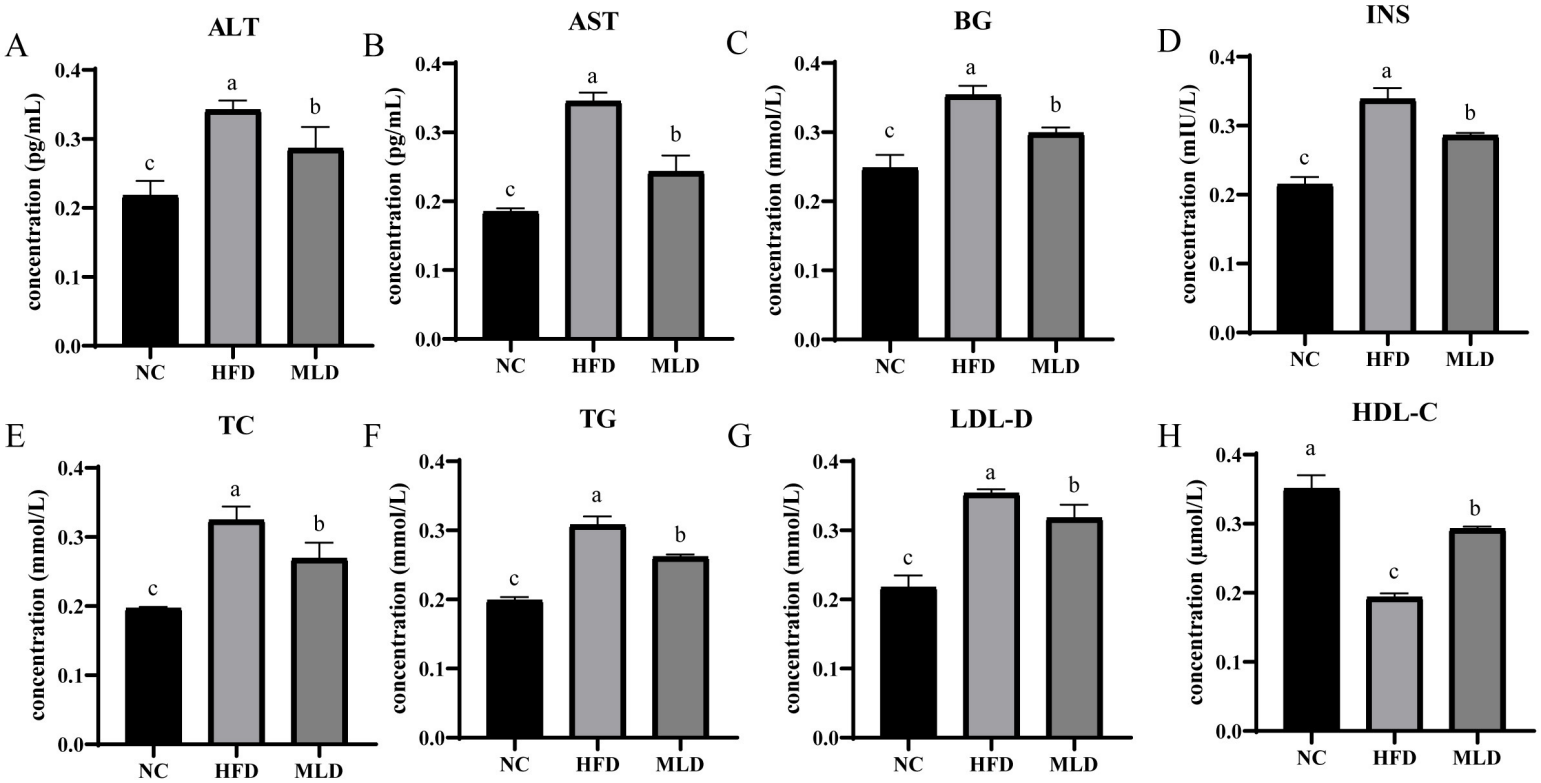
Effect of MLD on Insulin-Related Parameters and Serum Biochemical Markers in Mice Fed a High-Fat Diet. **(A)** Serum ALT, **(B)** Serum AST, **(C)** Serum BG, **(D)** Serum TG, **(E)** Serum TC, **(F)** Serum INS, **(G)** Serum HDL-C, **(H)** Serum LDL-C. Values are expressed as means ± SD, n=6. ELISA data were analyzed using one-way ANOVA followed by *post-hoc* Tukey's HSD test. Differences in lettering denote statistical significance (*p* < 0.05).

The biochemical analysis of MLD in mouse plasma is shown in Figure 2. ALT and AST levels are widely recognized as clinical biomarkers for liver injury (35). In mice fed a high-fat diet, both ALT and AST levels were significantly elevated (*p* < 0.05), indicating that the high-fat diet induced hepatic dysfunction. This is consistent with previous findings showing that HFD increased ALT and AST levels (36). Following MLD intervention, ALT and AST levels decreased by 16.4% and 29.6% (*p* < 0.05), respectively (Figures 2A, B), suggesting that MLD treatment can ameliorate liver dysfunction (37, 38). This is consistent with previous findings demonstrating that cooked red beans mitigate liver damage by reducing serum AST and ALT levels (39).

Lipids in the blood, including TG, TC, LDL-C, and HDL-C, serve as key indicators of lipid metabolism (38). Several studies have established a strong correlation between elevated TG levels and insulin resistance (40). The hypolipidemic effect of MLD in hyperlipidemic mice was assessed by quantifying serum levels of blood glucose (BG), insulin (INS), TC, TG, LDL-C, and HDL-C (Supplementary Table S2). High-fat diet-induced dyslipidemia was characterized by significantly elevated levels of BG (0.35 ± 0.03 vs. 0.25 ± 0.02), INS (0.34 ± 0.02 vs. 0.22 ± 0.02), TC, TG, and LDL-C (*p* < 0.05) (Figures 2C–G) and a marked reduction in HDL-C levels (*p* < 0.05) (Figure 2H). Following MLD intervention, serum levels of BG (0.30 ± 0.07 vs. 0.35 ± 0.03), INS (0.29 ± 0.01 vs. 0.34 ± 0.02), TC, TG, and LDL-C were reduced by 15.5%, 15.6%, 17.1%, 15.0%, and 10.1% (*p* < 0.05), respectively, whereas HDL-C levels increased by 51.1% (*p* < 0.05). These results suggest that MLD may mitigate dyslipidemia induced by a high-fat diet and help lower blood lipid levels. A rapid increase in blood glucose triggers the secretion of elevated insulin, which inhibits lipolysis and promotes fat synthesis, thereby contributing to the development of obesity. MLD feeding decreased insulin secretion, reduced fat accumulation in the liver, and effectively improved lipid regulation (41). This finding is consistent with a previous study, which showed that anacardic acid reduces hepatic fat accumulation by modulating serum biochemical markers (33).

### 3.5 Histological analysis

**Figure 3:**
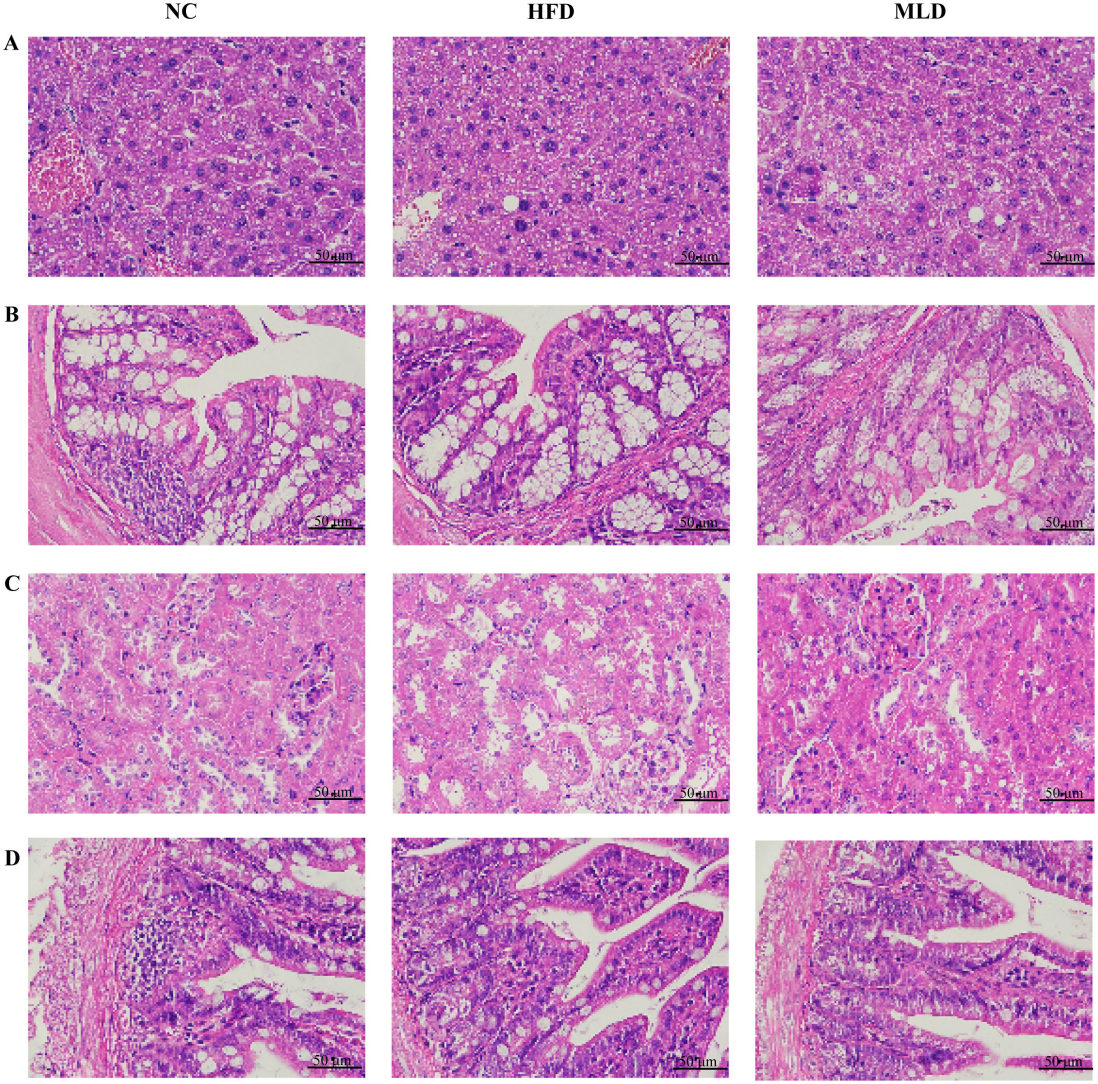
The effects of MLD on histopathological. Representative sections of immunohistochemically stained tissue sections from various organs with H&E are shown (scale bar, 50 μm). **(A)** Liver **(B)**, Colon **(C)**, Kidney **(D)**, and Small Intestine.

Both adipose tissue and the liver are essential metabolic and endocrine organs that play pivotal roles in the development of obesity (37). Figure 3 illustrates the results of H&E staining from various mouse tissues. Liver sections from the HFD group exhibited pronounced hepatocyte steatosis, characterized by the presence of rounded cytoplasmic vacuoles. In contrast, melanoidin intervention significantly alleviated hepatocyte degeneration, reduced both the size and content of adipocytes within the liver, and restored hepatocytes to a near-normal state (Figure 3A). Wu et al. investigated the effects of black garlic melanoidins on mice fed a high-fat diet and found that these compounds substantially reduced hepatic fat deposition and alleviated obesity-related fatty liver lesions (7). Colon tissue sections from all three groups exhibited normal morphology, characterized by deep crypts, a single-layer columnar epithelium on the surface, abundant intestinal glands, and no noticeable lesions (Figure 3B). Kidney tissue from the HFD group showed edematous degeneration in a small number of renal tubular epithelial cells, characterized by sparse, lightly stained cytoplasm and vacuolar degeneration with rounded vacuoles (Figure 3C). Small intestine tissue displayed normal morphology, characterized by well-defined intestinal villi on the surface of a single layer of columnar epithelium (Figure 3D). In conclusion, the H&E staining results suggest that melanoidins effectively reduce hepatocellular lipid accumulation induced by a high-fat diet.

### 3.6 Effects of MLD on gut microbiota

**Figure 4:**
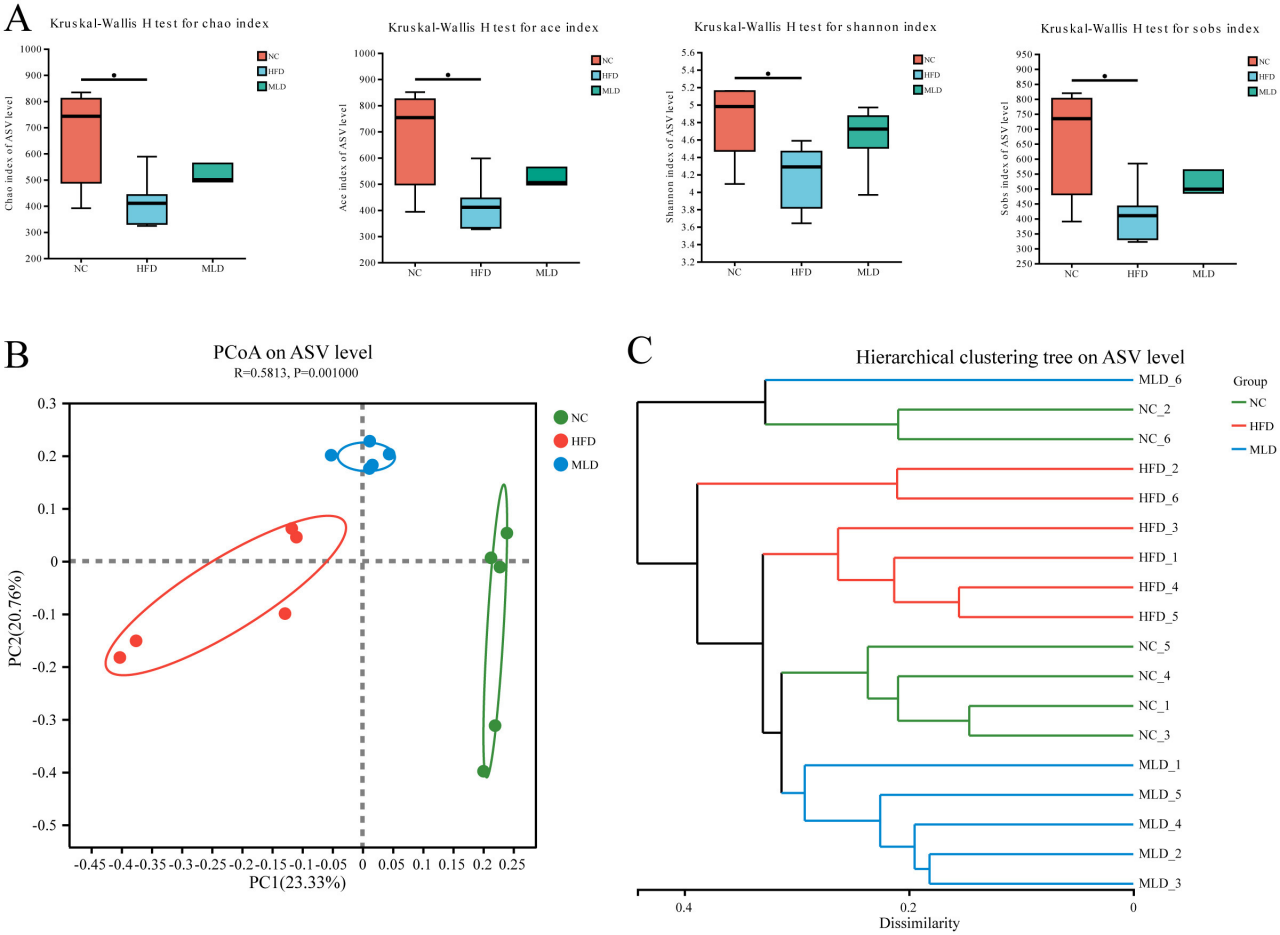
Effect of melanoidins on α and β diversity of gut microbiota. **(A)** α diversity index, **(B)** β diversity analysis, and **(C)** cluster analysis (*n* = 6, *P < 0.05).

After 20 weeks of gavage intervention, 16S rRNA gene sequencing was performed on bacteria extracted from the cecum of mice across all experimental groups. From the 16S rRNA high-throughput sequencing results, a total of 1,226,704, 506,457,392 bases of optimized sequences were obtained, with an average sequence length of 413 bp. Species annotation revealed the following taxonomic distribution: Domain: 1; Kingdom: 1; Phylum: 14; Class: 20; Order: 51; Family: 93; Genus: 175; Species: 278; ASV: 5,955. Sequences were clustered into OTUs based on 97% sequence similarity. To assess microbial community richness and diversity, α-diversity indices, including Chao1, Ace, Sobs, and Shannon, were calculated (38). The results showed that the HFD feeding significantly reduced the Chao1, Ace, and Sobs indices (Chao1: 410.02 vs. 742.56, Ace: 410.51 vs. 753.02, Sobs: 410 vs. 734; p < 0.05) (Figure 4A), reflecting a decrease in bacterial richness. Furthermore, the Shannon index, indicative of bacterial diversity, was notably lower in the HFD group compared to the NC group (4.29 vs. 4.98; p < 0.05), suggesting a reduction in both bacterial abundance and diversity. In the MLD treatment group, the Chao1 index was significantly lower than that in the NC group (499.95 vs. 742.56), but the Chao1, Ace, Sobs, and Shannon indices were all significantly higher in the MLD group compared to the HFD group (Chao1: 499.95 vs. 410.02, Ace: 504.58 vs. 410.51, Sobs: 498.5 vs. 410, Shannon: 4.72 vs. 4.29) (Figure 4A), indicating that melanoidin intervention partially reversed the decline in bacterial richness and diversity induced by the high-fat diet. The β-diversity of the mouse gut microbiota was evaluated using Principal Coordinates Analysis (PCoA) (42), with each point representing an individual sample within the same group. The color of each point corresponds to the respective group, providing a visual representation of group membership. The distance between two points reflects the degree of dissimilarity between the corresponding samples. The results showed a relatively large distance between samples from the NC and HFD groups (Figure 4B), indicating a significant difference in intestinal microbiota composition (*p* = 0.001). Treatment with MLD shifted the gut microbiota composition of the mice toward that of the NC group. Additionally, MLD treatment induced significant alterations in the gut microbiota composition of the mice, as demonstrated by the reduced distance between the MLD and NC groups along PC1. Cluster analysis further confirmed that samples from the MLD group clustered more closely with those from the NC group than with the HFD group (Figure 4C). These findings suggest that MLD treatment effectively ameliorates high-fat diet-induced gut dysbiosis and modulates the abundance and diversity of intestinal microbial communities, consistent with previous studies by Yang et al. (43).

The intestinal microbiota has been recognized as a critical factor in the onset and progression of obesity, with modulation of the microbiota emerging as a promising therapeutic strategy (44). The predominant microbial phyla in mice are *Bacteroidetes* and *Firmicutes*, both of which play crucial roles in metabolizing dietary fibers, carbohydrates, and proteins within the colon. Studies have demonstrated that the *Firmicutes-to-Bacteroidetes* ratio is increased in obese individuals, a shift that enhances the intestinal absorption of nutrients and calories (40). To investigate the impact of MLD treatment on gut microbiota structure, we examined microbial composition at both the phylum and genus levels. At the phylum level (Supplementary Figure S2A), HFD administration significantly altered the overall microbial composition compared to the NC group. Specifically, an increase in *Firmicutes* abundance was accompanied by a decrease in *Bacteroidetes* abundance. MLD treatment reverses this imbalance. These findings suggest that a high-fat diet induces disruption of the intestinal microbiota, whereas MLD treatment helps preserve intestinal microbial homeostasis. Higher *Firmicutes*-to-*Bacteroidetes* ratios correlate with obesity, while lower ratios are associated with improvements in obesity. Consistent with previous findings (45), our data revealed a 24.6% increase in this ratio in HFD mice compared to NC, while MLD treatment reduced the ratio by 38.9%. These findings support the potential anti-obesity effect of MLD through modulation of the *Firmicutes*-to-*Bacteroidetes* ratio.

At the genus level (Supplementary Figure S2B), marked shifts in the abundance of various bacterial taxa were observed. A significant increase in the abundance of *Blautia, Bacteroides, Roseburia, Helicobacter*, and *Erysipelatoclostridium* was observed in the HFD group, while notable decreases were detected in *norank_f_Muribaculaceae, norank_f_Oscillospiraceae, Lachnospiraceae_NK4A136_group, Alistipes, and Bifidobacterium*. MLD treatment effectively reversed these changes in microbial composition. This suggests that MLD has the potential to modulate gut microbiota dysbiosis in obese mice, restoring the microbial composition toward that observed in healthy controls. Notably, *unclassified_f_Lachnospiraceae*, typically elevated in high-fat diet models (46), showed only slight variation here, with no significant correlation to obesity, consistent with the findings of Li et al. (47). This may suggest that not all members of *Lachnospiraceae* are associated with obesity, highlighting the need for further investigation into bacterial variations at finer taxonomic levels (48). *Blautia, Roseburia*, and *Bacteroides*, which are associated with health, are key producers of short-chain fatty acids (SCFAs) (49, 50). However, excessive SCFAs production may exacerbate obesity (51). The HFD group may have contributed to obesity in mice by increasing the abundance of these genera, thereby promoting excessive SCFAs production. *Lachnospiraceae_NK4A136_group* produces acetate and butyrate (52), while *norank_f_Muribaculaceae* is a potential probiotic for propionate production (53). SCFAs promote gut health, improve insulin sensitivity, and help mitigate obesity and diabetes (54). Acetate and propionate inhibit hepatic lipid synthesis, while butyrate reduces blood glucose (36), which may explain the anti-obesity effects of MLD. These findings support research suggesting that *P. acidilactici* Y01 reduces obesity by modulating beneficial gut microbiota (55). Additionally, mitochondrial dysfunction is a key contributor to obesity (56). Previous studies have shown that *Bifidobacterium* promote mitochondrial biogenesis and function while improving lipid metabolism (57). This suggests a potential mechanism by which MLD prevents obesity through the regulation of *Bifidobacterium* abundance. In conclusion, diet-induced obesity is associated with elevated levels of glucose, lipids, and metabolic disorders. The mechanism by which MLD alleviates obesity may include reducing the *Firmicutes*-to-*Bacteroidetes* ratio, inhibiting harmful bacteria like *Erysipelatoclostridium* and *Bacteroides*, promoting beneficial bacteria such as *Bifidobacterium* and *norank_f_Muribaculaceae* to produce SCFAs, and enhancing mitochondrial biogenesis and adipose tissue function. These findings are consistent with previous studies in which mice were treated with bamboo shoots to modulate the gut microbiota and mitigate obesity (58).

### 3.7 Metabolomics analysis on protective effects of MLD

**Figure 5:**
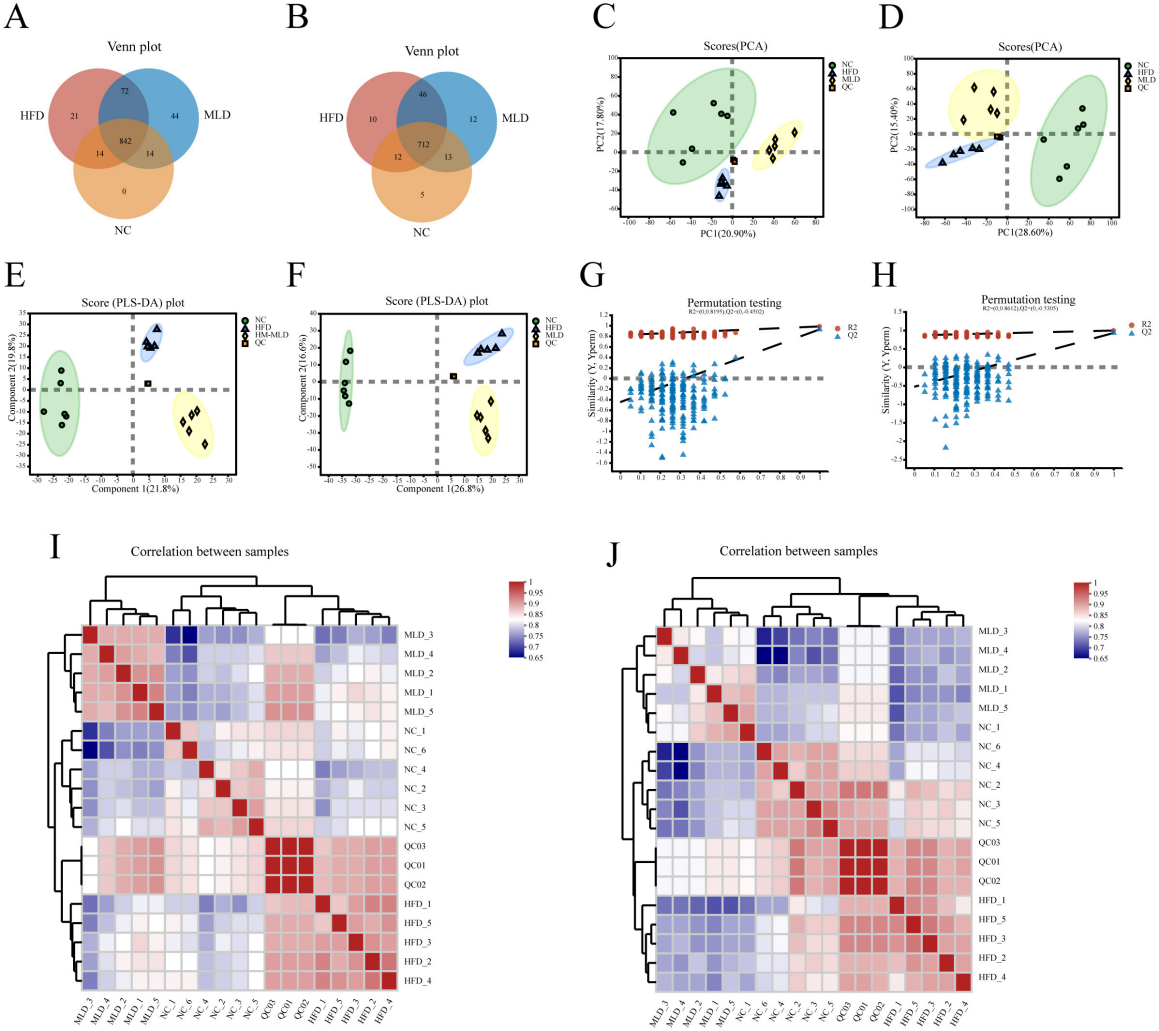
Differential analysis of metabolite expression across the NC, HFD, and MLD groups. **(A, B)** Venn plot, **(C, D)** PCA, **(E, F)** PLS-DA, **(G, H)** Permutation test of OPLS-DA mode, **(I, J)** Heatmap of sample correlation. *n* = 6.

**Figure 6:**
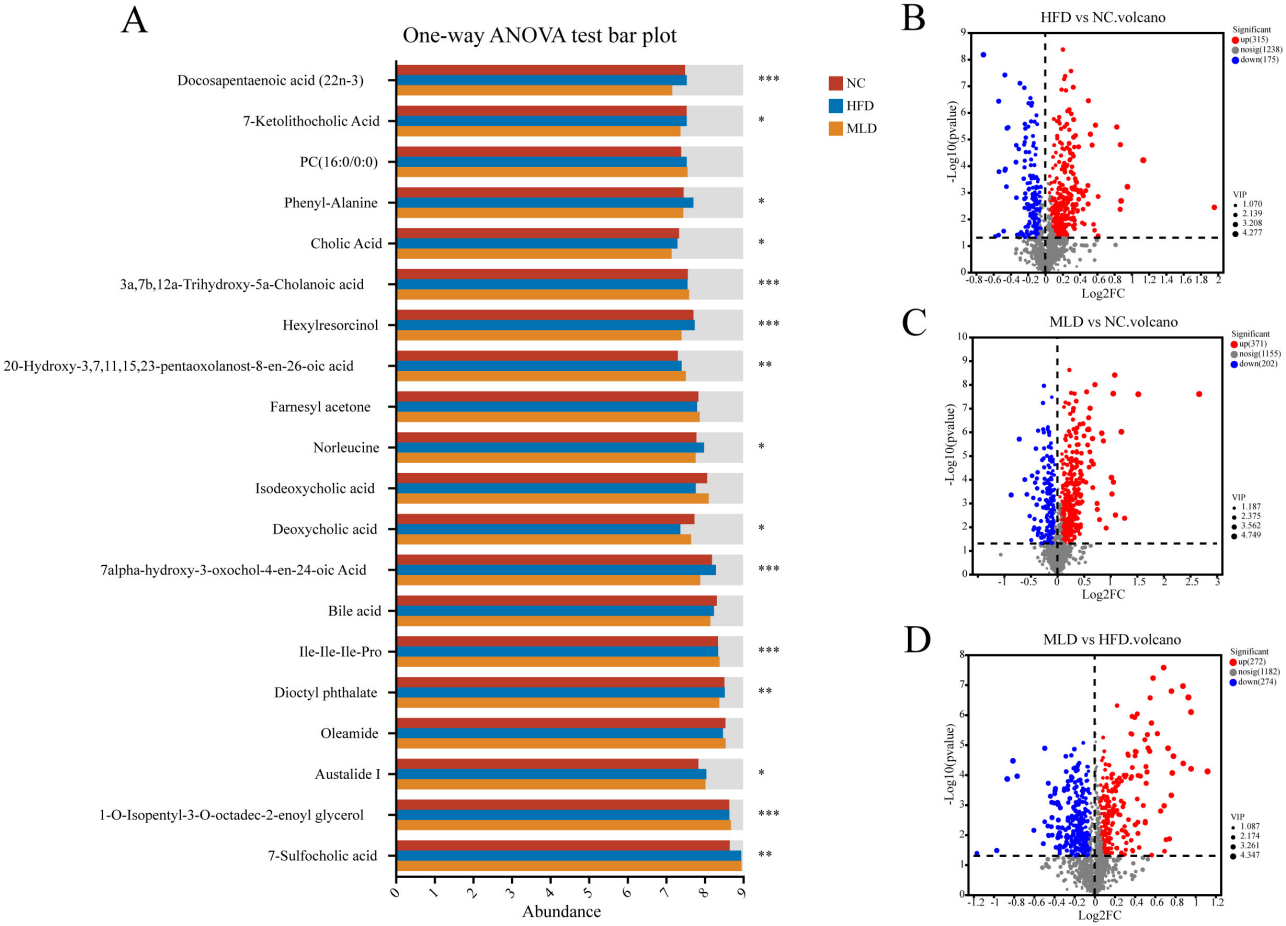
Differential metabolite analysis of NC, HFD, and MLD groups. **(A)** Multi-group comparative analyses, **(B)** HFD vs. NC Volcano, **(C)** MLD vs. NC Volcano, **(D)** MLD vs. HFD Volcano, with statistical significance indicated by asterisks (*n* = 6, *P < 0.05, **P < 0.01, ***P < 0.001), highlighting the distinct metabolic responses induced by MLD in the context of a high-fat diet.

Fecal samples from the NC, HFD, and MLD groups were analyzed using LC-MS untargeted metabolomics. Venn diagrams of positive and negative ions (Figures 5A, B) revealed significant differences in metabolite profiles among the three groups. Principal component analysis (PCA) demonstrated a clear separation of the NC group from the other two groups. The metabolite clusters of the MLD group were more similar to those of the NC group (Figures 5C, D). PLS-DA scatter plots further confirmed a distinct separation of the three groups, indicating significant differences in fecal metabolites (Figures 5E, F). The alignment analysis model parameters exhibited an increasing trend, suggesting the stability and accuracy of the model's predictions (Figures 5G, H). Heatmaps (Figures 5I, J) indicated strong correlations among the samples. Overall, both cluster analysis and PCA indicated substantial metabolic differences across the groups, with the most pronounced differences observed between the NC and HFD groups (59). In the two-ion model, a one-way ANOVA across multiple groups (Figure 6A) demonstrated significant differences in acid composition among the three groups. Differential metabolites were identified based on criteria of P < 0.05, VIP > 1, and FC > 1 or < 1 (Figures 6B–D). The results showed that the HFD group exhibited 490 differential metabolites, including 175 downregulated and 315 upregulated. In contrast, the MLD group exhibited 573 differential metabolites, comprising 202 downregulated and 371 upregulated metabolites. In contrast, the HFD and MLD groups exhibited a total of 546 differential metabolites, comprising 274 downregulated and 272 upregulated metabolites. This suggests that MLD treatment affected the metabolite content of mice on a high-fat diet (60). Ji et al. demonstrated that *Lomatogonium rotatum* altered the metabolic profile of mice on a high-fat diet, a finding that is in agreement with the results of the current study (40).

To further elucidate the metabolic pathways associated with MLD, differential metabolites were input into the KEGG database for pathway construction and analysis. The metabolite correlation heatmap (Supplementary Figures S3A, B) and correlation bubble plot (Supplementary Figures S3C, D) revealed distinct differences between the HFD and NC groups, as well as between the MLD and NC groups, indicating that MLD administration significantly altered the metabolic profile of the high-fat diet group. Metabolites with VIP values >4, such as Neomycin sulfate (VIP = 4.28), were identified as key biomarkers differentiating the HFD and NC groups (Supplementary Figure S3E). Moreover, metabolites, including Evoxine (VIP = 4.75), Coniferin (VIP = 4.32), Caryophyllene (VIP = 4.11), and Nivalenol (VIP = 4.46), were identified as biomarkers distinguishing between the MLD and NC groups (Supplementary Figure S3F). KEGG compound classification analysis (Supplementary Figures S3G, H) demonstrated an increase in lipid and steroid metabolites, accompanied by a decrease in carbohydrate and peptide metabolites in the MLD group after treatment. These findings are consistent with the results of Liu et al., which indicate that MLD has an impact on lipid metabolism (59). KEGG enrichment analysis (Supplementary Figures S3I, J) showed HFD enriched 16 metabolic pathways, notably lipid metabolism, amino acid metabolism, and organelle systems, while MLD enriched 18 pathways, emphasizing its effects on lipid and amino acid metabolism. KEGG topology analysis (Supplementary Figures S3K, L) revealed that glycerophospholipid metabolism had the most significant impact on the HFD group, consistent with prior studies (61). Fecal metabolite analysis revealed elevated glycerophospholipid levels in the HFD group, linked to fat accumulation and lipid dysregulation (62), indicating that HFD induces obesity primarily through the activation of glycerophospholipid metabolism. Arginine biosynthesis had the most significant impact on MLD, akin to findings linking ultra-processed foods to metabolic disorders (31). The expression levels of key metabolites enriched for MLD are presented in Supplementary Table S3. The arginine biosynthesis pathway was downregulated in the MLD group, consistent with studies showing that weight loss interventions similarly suppress this pathway (63). This suggests that MLD may function as a weight loss intervention by inhibiting arginine biosynthesis. However, previous studies have shown that arginine supplementation reduces fatty acid biosynthesis and enhances metabolic function (64). We assume that HFD induces abnormal arginine biosynthesis, driving metabolic disturbances that lead to obesity, while MLD may reduce obesity by downregulating this pathway to normalize arginine levels. However, further research is required to elucidate the underlying mechanism. Additionally, MLD downregulated sphingolipid metabolism pathways. This downregulation may serve as a compensatory mechanism, reducing Sphinganine 1-phosphate levels and potentially protecting the liver against further damage (45). In summary, MLD improves the metabolic dysregulation in obese patients by modulating the arginine biosynthesis and sphingolipid metabolism pathway, contributing to obesity reduction.

### 3.8 Potential correlation between gut microbiota and key metabolites

We performed a Spearman correlation analysis to evaluate the association between metabolite and microbiota data at the genus level, as depicted in Supplementary Figure S4. *Bacteroides* (*p* < 0.01), *Roseburia* (*p* < 0.05), and *Erysipelatoclostridium* (*p* < 0.05) exhibited significant positive correlations with 1-Pyrroline-5-carboxylic acid, while *Erysipelatoclostridium* was also significantly positively correlated with L-4-Hydroxyglutamate semialdehyde (*p* < 0.05). In contrast, *Muribaculaceae* showed a significant negative correlation with both 3-dehydroshikimate and ornithine (*p* < 0.05). 1-Pyrroline-5-carboxylic acid is involved in amino acid metabolism, whereas L-4-Hydroxyglutamate semialdehyde plays a role in arginine and proline metabolism, serving as an organic compound of L-alpha amino acids. Disruptions in amino acid metabolism have been implicated in the pathology of drug-induced liver injury (65). These results suggest that melanoidins may regulate amino acid metabolism by modulating the abundance of *Erysipelatoclostridium, Roseburia, Bacteroides*, and *Muribaculaceae*, potentially mitigating obesity and other metabolic disorders. In addition, the *g__Lachnospiraceae*_*NK4A136_group* was significantly negatively correlated with Sphinganine 1-phosphate, a key metabolite in sphingolipid metabolism. Sphingolipid metabolites are critical regulators of numerous physiological and pathophysiological processes (66). Specifically, sphingosine 1-phosphate is recognized for its antiapoptotic and endothelial cell-protective effects, which mitigate ischemia-reperfusion injury and inflammation (67). Additionally, sphingosine 1-phosphate may enhance insulin sensitivity and reduce the risk of cardiovascular disease associated with weight loss and exercise training (68). These findings suggest that melanoidins may influence sphingolipid metabolism by modulating the abundance of *g__Lachnospiraceae_NK4A136_group*. Hou et al. (31) demonstrated that ultra-processed food (UPF) intake can impact liver health by disrupting metabolic processes through alterations in the gut microbiota. In summary, MLD may alleviate metabolic disorders by regulating both amino acid and sphingolipid metabolism through the modulation of key bacterial genera, thereby acting as a potential anti-obesity agent.

### 3.9 Research prospects and limitations

Our findings suggest that black jujube MLD supplementation effectively improves lipid metabolism and reshapes the gut microbiota in obese mice. While MLD mitigates obesity development, several limitations must be addressed: (1) Although MLD shows promising potential in obesity prevention, the effects of physical activity and energy expenditure on obesity and metabolism remain unexplored. These factors require further investigation in future studies. (2) While MLD appears to protect the liver by reducing ALT and AST levels, additional research is needed to clarify the mechanisms underlying hepatic inflammation and oxidative stress. (3) Our analysis of metabolism pathways, gut microbial diversity, and their interrelations is preliminary. Correlations between fecal metabolites and specific microbial communities do not establish causality. To establish causal relationships, future studies using fecal microbiota transplantation are recommended. (4) Although obesity affects both genders, the influence of the female endocrine system on disease progression remains underexplored. Most studies have focused on males, and future research should examine gender differences in obesity prevention with MLD supplementation. Despite these limitations, our data provide preliminary evidence that black jujube MLD may prevent obesity through modulation of amino acid and sphingolipid metabolism, as well as gut microbiota.

## 4 Conclusions

This study reveals the structure of melanoidins and investigates the effects of MLD on high-fat diet-induced obesity and associated metabolic disorders in mice, thereby complementing the potential role of black jujube melanoidins in preventing the development of obesity. The study demonstrated that the high-fat diet significantly increased body weight and adiposity, indicating metabolic disturbances when compared to the NC group. MLD effectively reversed these changes without altering energy intake. The OGTT and ITT demonstrated that MLD stabilized blood glucose levels, reduced body weight, and regulated insulin and glucose homeostasis in obese mice. Moreover, serum biochemical analysis revealed that MLD intervention alleviated high-fat diet-induced liver injury and dyslipidaemia, exerting a hypolipidemic effect. Histological analysis, as determined by H&E staining, revealed that MLD improved liver health markers and reversed high-fat diet-induced fat accumulation in hepatocytes. This effect may be mediated by alterations in the gut microbiota, restoring the impaired gut ecology to a healthy genus-level state. This includes reducing the *Firmicutes*-to-*Bacteroidetes* ratio, suppressing harmful bacteria such as *Erysipelatoclostridium* and *Bacteroides*, and enhancing the abundance of beneficial bacteria like *Bifidobacterium, norank_f_Muribaculaceae*, and others. Additionally, MLDs may alleviate metabolic disorders by downregulating arginine and sphingolipid metabolism, potentially through the modulation of key microbial genera. These findings suggest that MLDs could mitigate obesity and related conditions by influencing the gut microbiota-metabolism axis.

## Data Availability

The original contributions presented in the study are included in the article/Supplementary material, further inquiries can be directed to the corresponding author.
