# Supplementary material for: Modulatory effects of black jujube melanoidins on gut microbiota and metabolic pathways in high-fat diet-induced obesity
Source: Front Nutr. 2025 May 6;12:1580439. doi: 10.3389/fnut.2025.1580439 (PMC12088948; doi:10.3389/fnut.2025.1580439)
Supplement: Supplementary file 1 [file Table_1.docx]

Supplementary Material

# Supplementary Figures and Tables

## Supplementary Figures


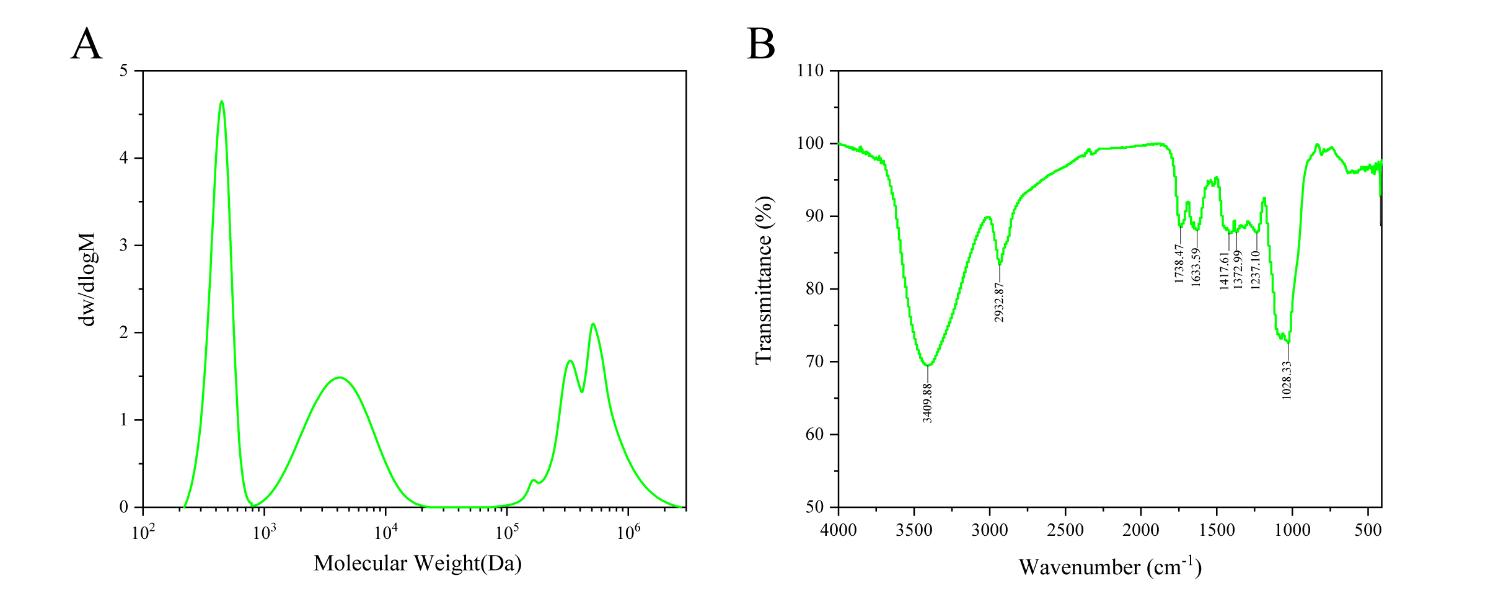


Supplementary Figure S1 Molecular weight distribution (A) and FTIR (B) of melanoidins.


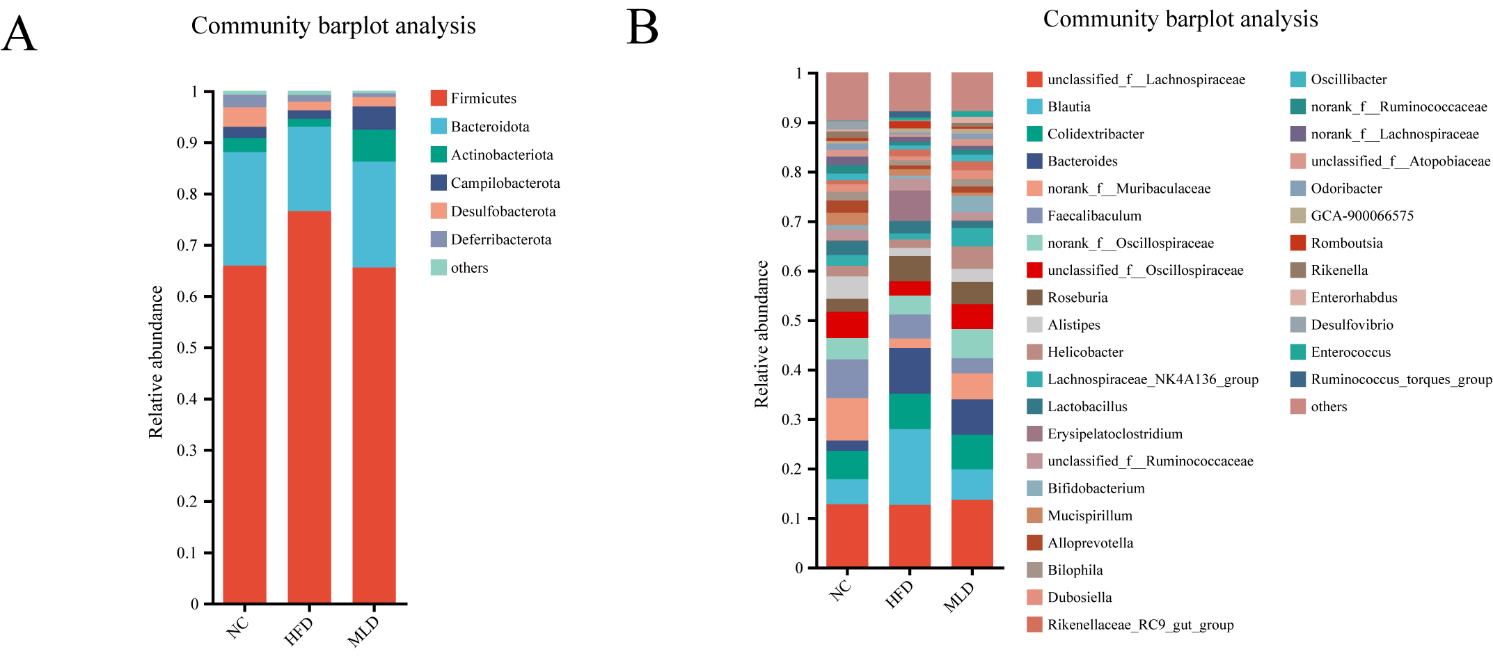


**Supplementary Figure S2.** Analysis of community composition at the phylum and genus level. (A) Phylum level community composition; (B) Genus level community composition. n=6.


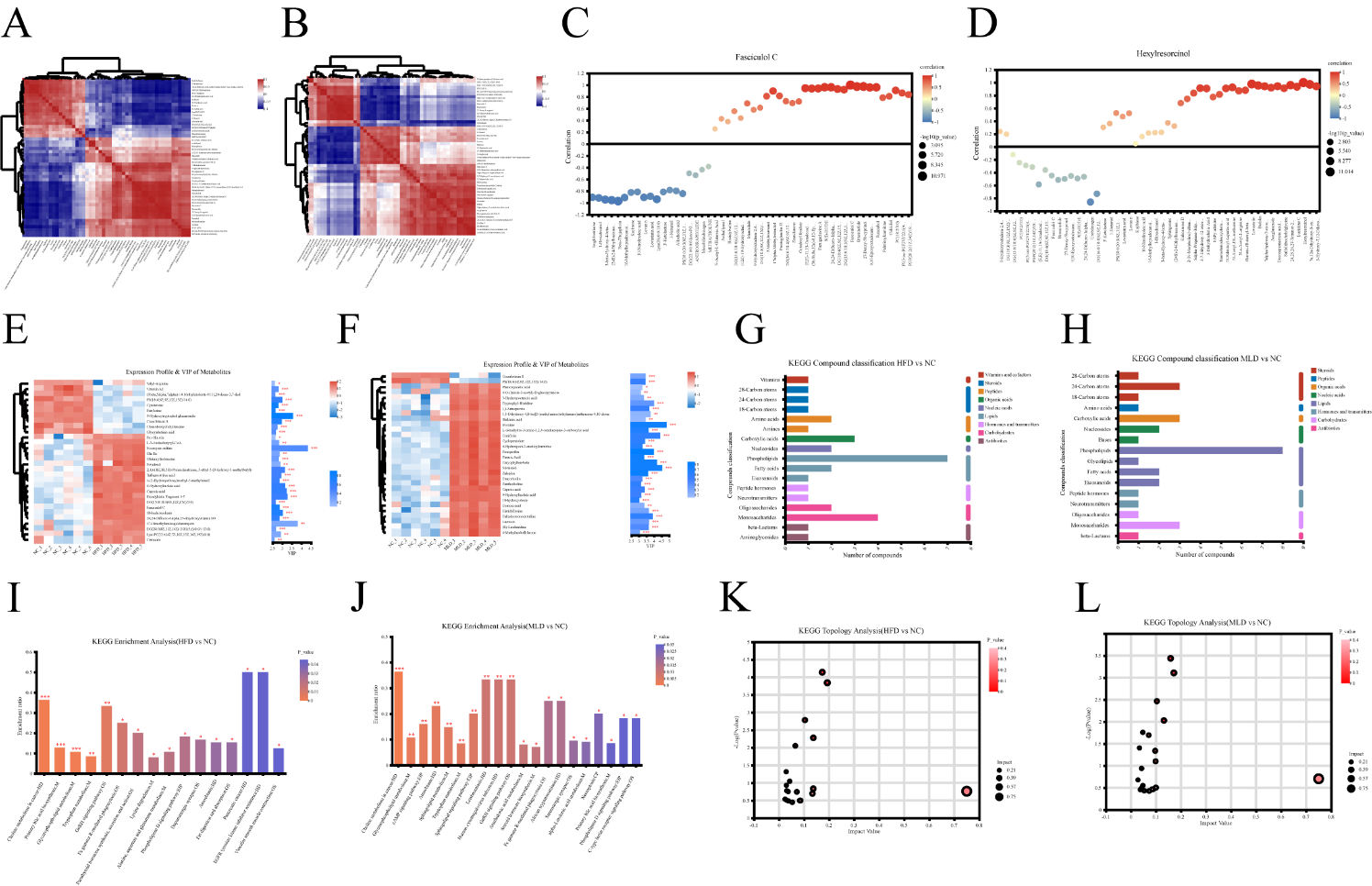


**Supplementary Figure S3.** Metabolic set analysis of the three groups NC, HFD, and MLD. (A, B) correlation analysis; (C, D) Correlation bubble chart; (E, F) VIP values analysis; (G, H) KEGG compound classification analysis; (I, J) KEGG enrichment analysis; (K, L) KEGG topology analysis.


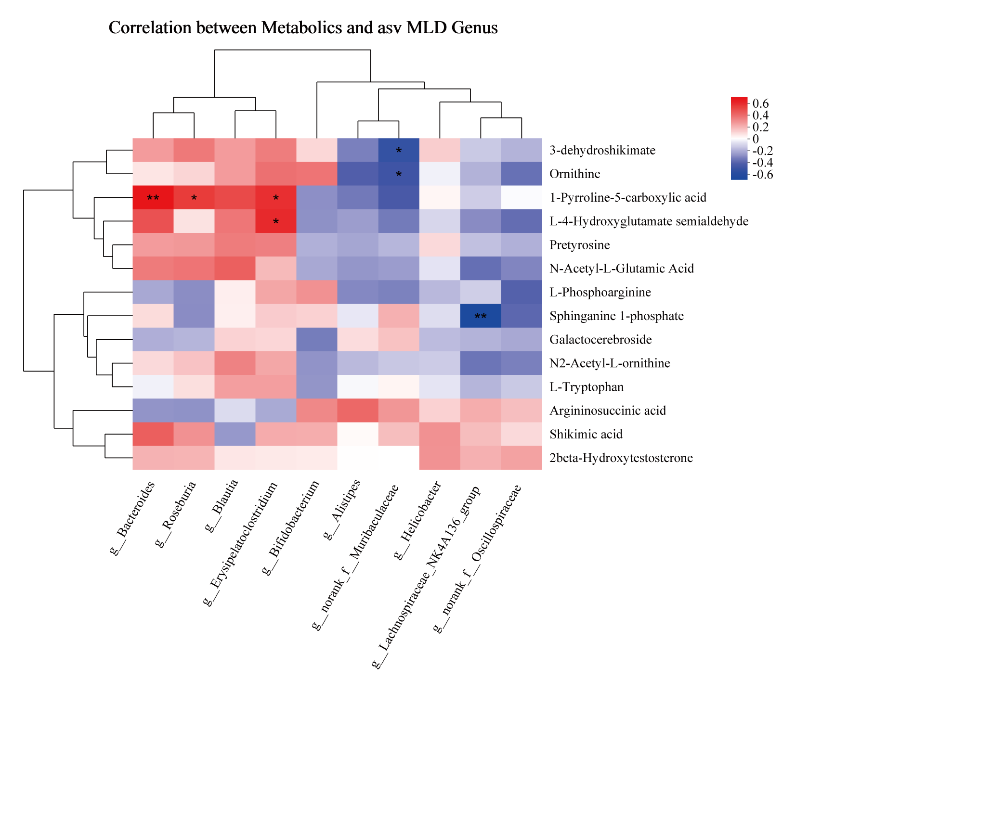


Supplementary Figure S4 Spearman correlation analysis of gut microbiome and metabolomics. (n=6, **p<0.01, *p<0.05)

## Supplementary Tables

**Supplementary Table S1.** Dose table of mice by gavage

| Groups | Feed lipid content (%) | Gavage treatment (13-20 weeks) |
| --- | --- | --- |
| Normal feed control group (n=12) | 10% | Equal volume of saline |
| High-fat diet group (n=12) | 45% | Equal volume of saline |
| melanoidin group (n=12) | 45% | 500 mg/kg MLD |

Supplementary Table S2. Changes in Serum ALT, AST, BG, INS, TC, TG, LDL-C and HDL-C

| Groups | NC | HFD | MLD |
| --- | --- | --- | --- |
| ALT (pg/mL) | 0.22±0.02^c^ | 0.34±0.01^a^ | 0.29±0.03^b^ |
| AST (pg/mL) | 0.19±0.01^c^ | 0.35±0.01^a^ | 0.24±0.02^b^ |
| BG (mmol/L) | 0.25±0.02^c^ | 0.35±0.03^a^ | 0.30±0.07^b^ |
| INS (mIU/L) | 0.22±0.02^c^ | 0.34±0.02^a^ | 0.29±0.01^b^ |
| TC (mmol/L) | 0.20±0.01^c^ | 0.32±0.02^a^ | 0.27±0.02^b^ |
| TG (mmol/L) | 0.20±0.01^c^ | 0.31±0.01^a^ | 0.26±0.01^b^ |
| LDL-C (mmol/L) | 0.22±0.02^c^ | 0.35±0.01^a^ | 0.32±0.02^b^ |
| HDL-C (μmol/L) | 0.35±0.02^a^ | 0.19±0.01^c^ | 0.29±0.01^b^ |

All values are expressed as mean ± SD. The different letters in a row represented significant differences at p < 0.05.

Supplementary Table S3 Screening results of differential metabolites

| Groups | Mode | Formula | VIP | FC | P_value | Regulate |
| --- | --- | --- | --- | --- | --- | --- |
| 3-dehydroshikimate | pos | C7H7O5- | 1.1945 | 0.9057 | 0.09285 | down |
| Ornithine | pos | C5H12N2O2 | 1.2749 | 0.9304 | 0.01282 | down |
| 1-Pyrroline-5-carboxylic acid | neg | C5H7NO2 | 1.2630 | 0.9420 | 0.01108 | down |
| L-4-Hydroxyglutamate semialdehyde | neg | C5H9NO4 | 1.4707 | 0.9391 | 0.003476 | down |
| Pretyrosine | neg | C10H13NO5 | 1.1534 | 0.9442 | 0.02959 | down |
| N-Acetyl-L-Glutamic Acid | neg | C7H11NO5 | 2.0056 | 0.8892 | 0.005737 | down |
| L-Phosphoarginine | neg | C6H15N4O5P | 2.2040 | 0.8272 | 0.01307 | down |
| Sphinganine 1-phosphate | pos | C18H40NO5P | 0.8106 | 0.9436 | 0.2276 | down |
| Galactocerebroside | pos | C25H47NO9 | 2.2974 | 0.8113 | 0.0003345 | down |
| N2-Acetyl-L-ornithine | pos | C7H14N2O3 | 1.6462 | 0.9159 | 0.000344 | down |
| L-Tryptophan | neg | C11H12N2O2 | 1.4307 | 0.9388 | 0.01738 | down |
| Argininosuccinic acid | pos | C10H18N4O6 | 1.2527 | 1.0914 | 0.0412 | up |
| Shikimic acid | pos | C7H10O5 | 2.4478 | 1.617 | 0.03498 | up |
| 2beta-Hydroxytestosterone | neg | C19H28O3 | 1.0677 | 1.0677 | 0.0794 | up |
